# Abdominal Cyst, Goitre, Hip Amputation

**Published:** 1915-04

**Authors:** Robert T. Morris

**Affiliations:** New York. Professor of Surgery, N. Y. Post. Grad. Medical School. 616 Madison Avenue


### BUFFALO MEDICAL JOURNAL

Yearly Volume 70. APRIL, 1915 No. 9

### ORIGINAL ARTICLES

The right is reserved to decline papers not dealing with practical medical and surgical subjects, and such as might offend or fail to interest readers. Contributors are solely responsible for opinions, methods of expression and revision of proof.

### Abdominal Cyst, Goitre, Hip Amputation. Report of a Clinic

By ROBERT T. MORRIS, M. D., New York.

Professor of Surgery, N. Y. Post. Grad. Medical School,
March 3, 1915.

#### Abdominal Cyst.

Our first patient, a woman fifty-one years of age, states that for some years she has noticed a gradually enlarging mass below the navel, situated nearly in the middle line. Recently this has begun to cause distress.

The patient says that I operated upon her nine years ago, but that record cannot be found at the hospital. Following the line of the former incision, extending from the pubis to near the navel, I find that the abdominal wall was completely repaired after the former operation, and there is no sign of defect.

Within the peritoneum we come to a mass about the size of a cocoanut, adherent to everything in general and to nothing in particular. This is shelled out **en masse**, and upon being opened is found to be filled with a thick "pea-soup fluid." The nature of this tumor we may not know until a report has been received from laboratory. The patient's uterus and adnexa were evidently removed at my former operation, and in all probability this cyst is the degenerated remains of an ovarian graft, as I was employing such grafts frequently, for the purpose of avoiding precipitate menopause, nine years ago. Pathologists report confirms the idea that the tumor is a degenerated ovarian graft.

### Exophthalmic Goitre.

Our next patient, a young woman in great distress, with protruding eyes and extremely rapid heart action, gives the familiar picture of a case of exophthalmic goitre. It is difficult to draw the line arbitrarily between different kinds of goitre, because these merge into each other by insensible gradations. It is better perhaps, to speak of cases as representing hyperthyroidism or hypothyroidism. In any event the most important feature is the one of abnormal increase or decrease of thyroid gland secretion. Incidentally almost all of the other ductless glands in the body have some sort of relation to thyroid disturbance.

In former days the phrenologist ascribed all human characteristics to certain bumps upon the skull, but when it was pointed out that an equal number of bumps might be found upon the base of the skull, he was at a loss to find a new lot of characteristics for these bottom bumps. Perhaps these bottom bumps represent our different kinds of dual nature. The point which I wish to make is that the phrenologist when ascribing all characteristics to bumps which he could feel, was no more superficial in his grasp of the subject, than is the doctor who looks only upon the thyroid gland as the chief malefactor in a case in which a thyroid gland happens to be the one most clearly in sight.

Most of the cases of hyperthyroidism or hypothyroidism belong to the internist, rather than to the surgeon, but occasionally we find a case in which surgical treatment gives marked temporary benefit. In this present instance, the young woman is suffering so intensely that we must give her quick relief and I shall remove the right thyroid lobe by the "safety first" method. This is a method which you have seen me employ many times recently. It is not so attractive as some of the other operations, but is a very safe one.

The patient has been anaesthetized lightly by rectal anaesthesia and the anaesthesia is continued with nitrous oxide and oxygen. The patient did not know that she was to have an operation performed, and went under the influence of the ether in the rectum without having a suspicion of what we were about to do.

The skin over the line of incision is locally anaesthetized with novocaine, and the right gland of the thyroid is exposed. The essential part of the "safety first" operation is now introduced. It consists in splitting the fascial layers of the capsule down to the gland structure proper and brushing away all layers of the capsule with a gauze wipe. This leaves the parathyroids and the blood vessels practically intact. The substance of the gland of the right lobe is now removed by **morcellement** with a pair of strong forceps. All internal secretion which may have escaped in this process is carefully flushed out of the wound with salt solution and the wound is packed with balsam Peru gauze for the purpose of controlling oozing. The wound is now sutured over this gauze, leaving one end protruding for drainage. In two or three days we shall cut the sutures and then allow the gauze to be thrown out when it is loosened by granulation. The wound walls will then be pressed together for secondary adhesion and we shall trim the skin margin and the cut edges of the platysma myoides muscle for the purpose of making a nice scar. This division of the operation into two steps constitutes the only disagreeable feature, but the patients have so little disturbance and the operation is so easily done that I am very much pleased with the ultimate results.

### Hip Amputation.

Our patient, a fully developed and apparently well nourished man, gives a history of diabetes of recent development. Diabetes mellitus is nothing but a symptom, and when this symptom is presented it means that we are always to go to work and find out just what does really ail the patient. Sometimes a toxic influence from the colon or toxic influence from the bowel will cause a pancreatitis with consequent squeezing of the islands of Langerhans. Sometimes there is irritation of the floor of the fourth ventricle of the brain from any one of several causes. I will not go into this large subject now, because the point which interests us immediately is the fact that the patient has rapidly progressing gangrene of almost all of the right leg, extending nearly to the hip.

The patient has received a spinal injection of two ampoules of 10% novocaine solution. It was an American physician, Corning, you will remember, who introduced this method. We next anaesthetize the skin, cutting off the nociceptor influence by injecting the skin along the lines of proposed incision with 1% novocaine solution. The idea of cutting off the impulse from nociceptor nerves relieves the patient's centres of consciousness from any undue degree of shock. This resource belongs to another American physician please remember, Dr. Crile. We now with a pair of scissors neatly expose the sciatic nerve, and the anterior crural nerves. These are injected with 1% novocaine solution. The latter resource was introduced by another American physician Dr. Cushing. It blocks the nerves in such a way that harmful impulse is not transmitted to the centres of consciousness.

Our next step consists in ligating the femoral artery and vein at the site of Poupart's ligament. Everything is now ready for the main part of the operation. You observe that the patient has shown no sign of discomfort whatsoever, for he has been conversing with us during all this part of the operation in a normal way.

I now with a large knife make a sweeping incision, cutting skin and muscles for shaping the flaps. Our patient happened to be looking around over the audience while this was being done, and you perceived that he was unaware of what in former days was a shocking procedure. The femur is divided close to the great trochanter and the operation is completed with the exception of picking up some small bleeding vessels and applying the dressing.

The nurse who kept her hand upon the patient's pulse during the entire operation states that there was very little disturbance at any period of the operation. It was my intention to have a blood pressure apparatus ready to register any change in blood pressure during the various stages of the operation, but at the last moment this was forgotten. It must have been evident to all of you that this patient not only suffered no shock from the operation, but that during its entire course he spoke with us as one assistant might speak to another and was interested in looking about over faces in the audience. In reply to my question if he felt any pain he now says, "No, but there was a little feeling like a burning at one time." Such an operation as this would have astounded surgeons of a very few years ago, and yet today the avoidance of shock in a case of this sort has been made possible by three American physicians, Corning, Cushing and Crile.

616 Madison Avenue.

---

**Relationship of Gastric Ulcer to Gastric Carcinoma.** Louis B. Wilson and Ivan E. McDowell, "Am. Jour. of the Med. Sci., Dec., 1914. Of 399 cases of operated gastric cancer, examination of the primary lesion showed ulcers in 57.6%. In 46 cases of gastric cancer with specimens obtained at necropsy, only 38 per cent showed positive evidence of ulcer. While the examinations did not necessarily indicate pre-existing ulcer, the history usually indicated this condition and the authors conclude that gastric cancer rarely develop except at the site of a pre-existing ulcer. (Note: The general opinion may be expressed that while, looking backward from the standpoint of existing cancer, ulcer is usually more or less indicated by microscopic evidence and symptomatology in the history, comparatively few ulcers, treated as such, either medically or surgically, even develop cancer. Yet, in medical experience, gastric cancer is considerably more prevalent than ulcer.

